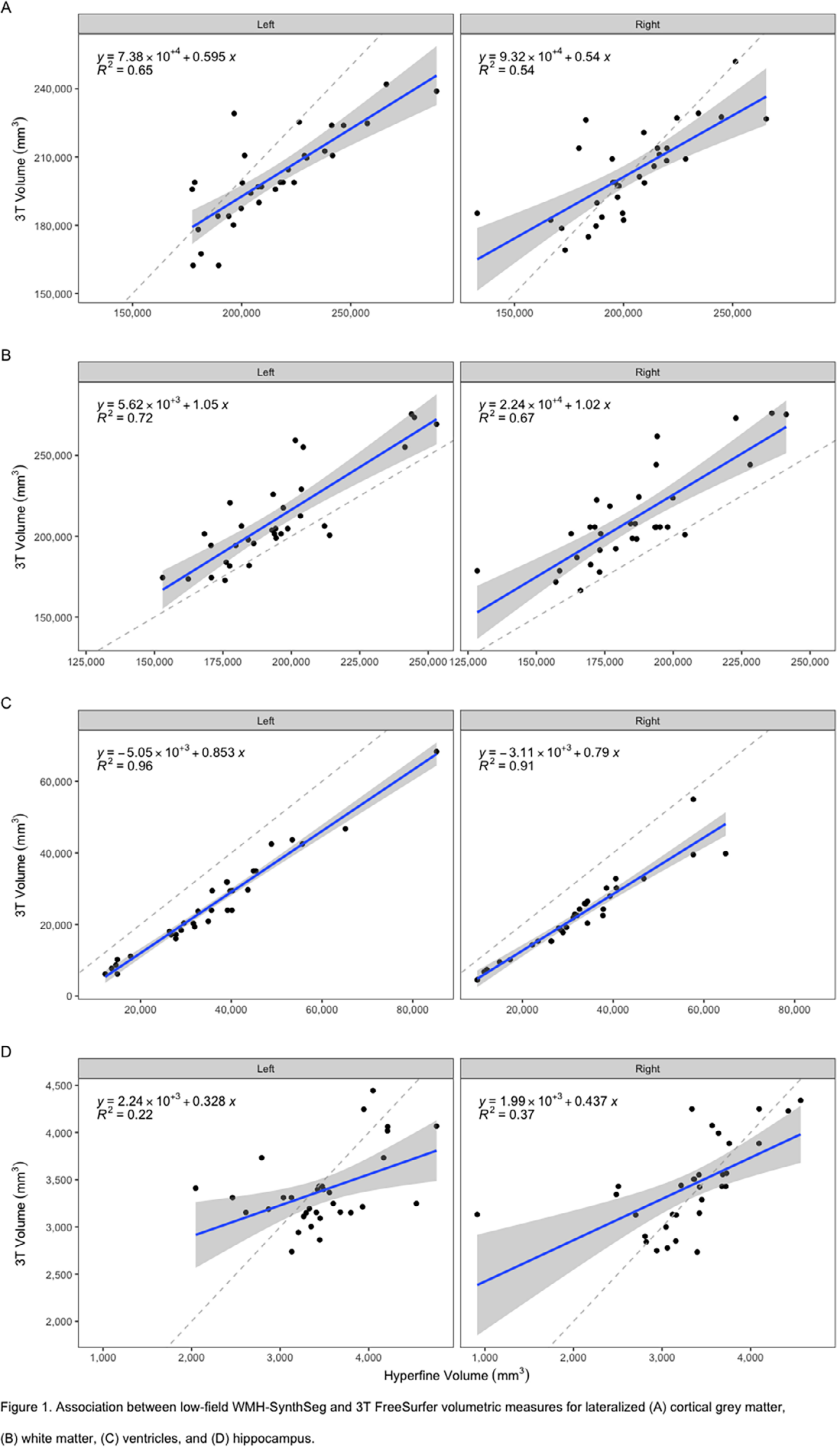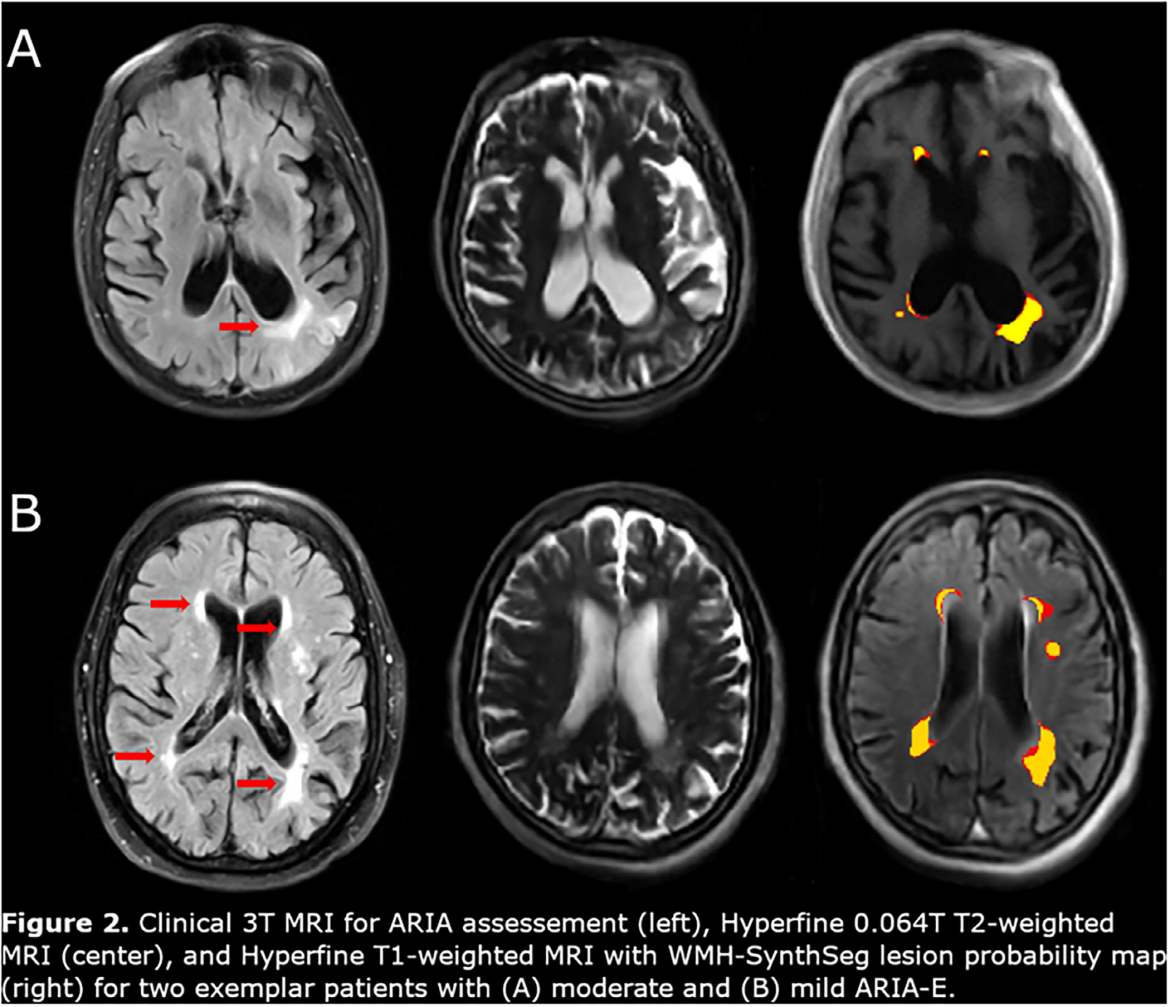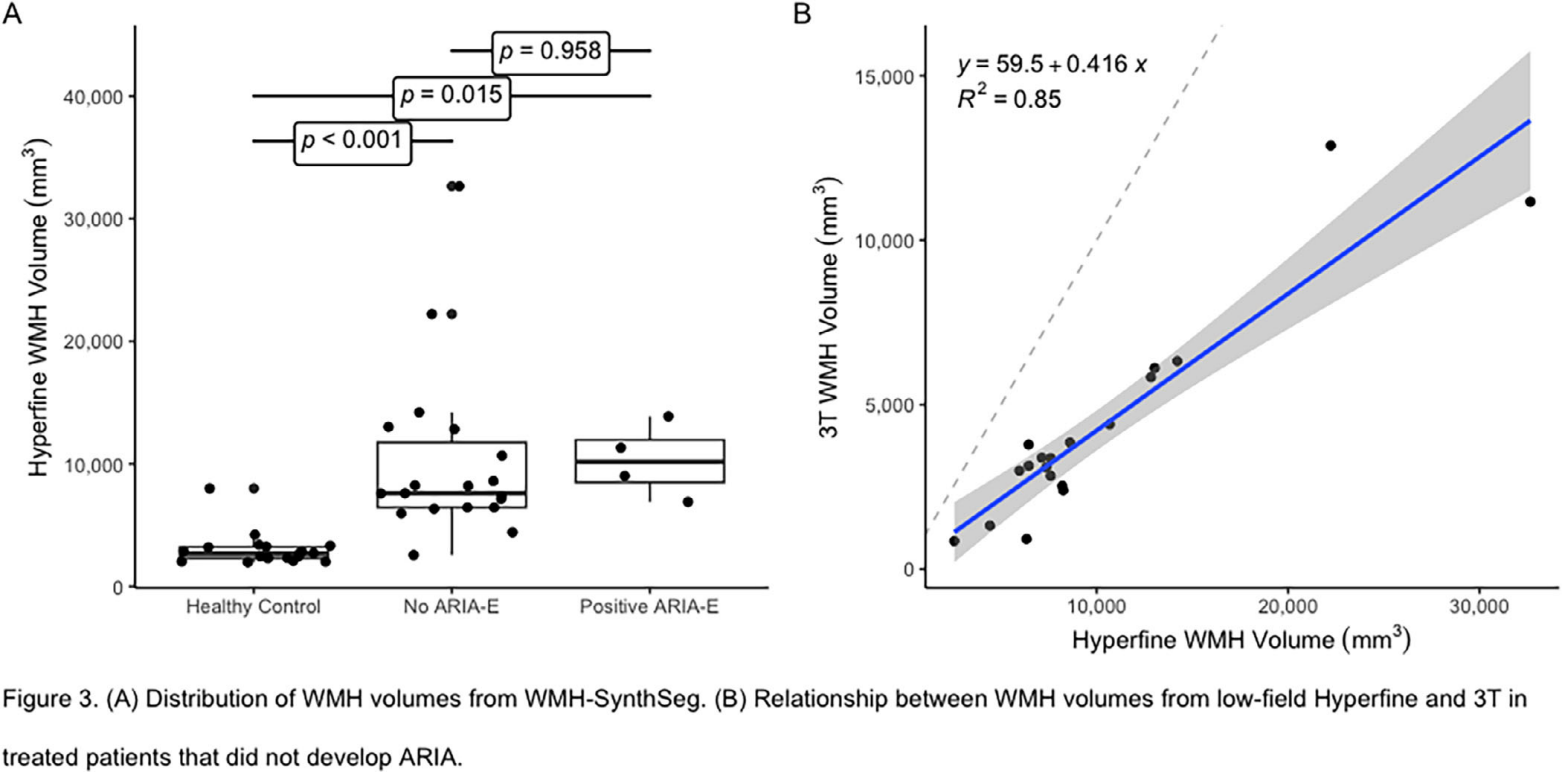# Evaluating Volumetric and White Matter Hyperintensity Lesion Measures for Low‐field MRI using WMH‐SynthSeg

**DOI:** 10.1002/alz70856_106017

**Published:** 2026-01-09

**Authors:** Hope Shimony, Sarah J. Keefe, Kavon J Sharifi, Jude‐Patrick Nnamdi Okafor, Nelly Joseph‐Mathurin, Jessica N Banks, Jessica Hu, Stephen Jarman, Edmond Knopp, Madeline Paczynski, Joo Lim, Alexandra Venuto, Nupur Ghoshal, B. Joy Snider, Shaney Flores, Tammie L.S. Benzinger

**Affiliations:** ^1^ Washington University School of Medicine, St. Louis, MO, USA; ^2^ Washington University in St. Louis, St. Louis, MO, USA; ^3^ Hyperfine, White Plains, NY, USA; ^4^ Knight Alzheimer Disease Research Center, St. Louis, MO, USA; ^5^ Washington University School of Medicine in St. Louis, St. Louis, MO, USA

## Abstract

**Background:**

White matter hyperintensities (WMH) are known predictors of amyloid‐related imaging abnormalities (ARIA) in patients undergoing anti‐amyloid immunotherapy (AAT) for Alzheimer disease. WMHs and brain volumetric changes can potentially be captured by low‐field magnetic resonance imaging (MRI) that imposes minimal safety risks to those with contraindicators to high‐field MRI. We investigate the recently released FreeSurfer WMH‐SynthSeg for volumetric and WMH lesion processing of low‐field MRI and its comparability to 3T MRI.

**Method:**

Low‐field head MRI scans for 19 healthy controls and 23 patients undergoing AAT were acquired on a 0.064T Hyperfine SwoopÒ Portable MRI scanner. Treated patients also underwent a 3T MRI for ARIA screening by a clinical neuro‐radiologist per treatment protocol. Volumetric measures of cortical grey matter, white matter, ventricles, and hippocampus were extracted from WMH‐SynthSeg for low‐field and FreeSurfer‐7.4 for 3T MRI. WMH lesion probability maps were generated for all participants from WMH‐SynthSeg. Linear regressions examined agreement between volumetrics for low‐field and 3T MRI in treated patients without ARIA. Spatial localization of WMH lesions with ARIA pathology were visually assessed and compared with the clinical 3T scan. Finally, WMH volume measures were assessed amongst healthy controls and patients with and without ARIA using a pairwise Wilcoxon with Benjamini‐Hochberg correction for multiple comparisons.

**Result:**

Four patients were clinically identified as having ARIA with cerebral edema (ARIA‐E) from 3T MRI. WMH‐SynthSeg estimates of lateral ventricular, white matter, and cortical grey matter volumes agreed with 3T MRI but tended to be significantly underestimated for the hippocampus (Figure 1). WMH lesion probability maps aligned with known ARIA but failed to capture the entire area affected (Figure 2). While WMH volumes for healthy controls were significantly lower than those on AAT therapy, WMH volumes were not significantly different between AAT patients with and without ARIA‐E (Figure 3).

**Conclusion:**

WMH‐SynthSeg provides comparable volumetric measures to 3T for large brain regions and can spatially capture known ARIA. However, in our small sample, these measures were not sensitive enough to fully identify areas of ARIA. Further development is needed to improve small region quantification and WMH lesion detection, particularly at low‐fields.